# How Do Grass Species, Season and Ensiling Influence Mycotoxin Content in Forage?

**DOI:** 10.3390/ijerph10116084

**Published:** 2013-11-12

**Authors:** Jiri Skladanka, Vojtech Adam, Petr Dolezal, Jan Nedelnik, Rene Kizek, Hana Linduskova, Jhonny Edison Alba Mejia, Adam Nawrath

**Affiliations:** 1 Department of Animal Nutrition and Forage Production, Faculty of Agronomy, Mendel University in Brno, Zemedelska 1, Brno CZ-613 00, Czech Republic; E-Mails: dolezal@mendelu.cz (P.D.); jeam1604@gmail.com (J.E.A.M.); adam.nawrath@mendelu.cz (A.N.); 2 Department of Chemistry and Biochemistry, Faculty of Agronomy, Mendel University in Brno, Zemedelska 1, Brno CZ-613 00, Czech Republic; E-Mails: vojtech.adam@mendelu.cz (V.A.); kizek@sci.muni.cz (R.K.); 3 Central European Institute of Technology, Brno University of Technology, Technicka 3058/10, Brno CZ-616 00, Czech Republic; 4 Research Institute for Fodder Crops, Ltd. Troubsko, Zahradni 1, Troubsko CZ-664 41, Czech Republic; E-Mails: nedelnik@vupt.cz (J.N.); moravcova@vupt.cz (H.L.)

**Keywords:** grass, silage, mycotoxin, environmental factor

## Abstract

Mycotoxins are secondary metabolites produced by fungal species that have harmful effects on mammals. The aim of this study was to assess the content of mycotoxins in fresh-cut material of selected forage grass species both during and at the end of the growing season. We further assessed mycotoxin content in subsequently produced first-cutting silages with respect to the species used in this study: *Lolium perenne* (cv. Kentaur), *Festulolium pabulare* (cv. Felina), *Festulolium braunii* (cv. Perseus), and mixtures of these species with *Festuca rubra* (cv. Gondolin) or *Poa pratensis* (Slezanka). The mycotoxins deoxynivalenol, zearalenone and T-2 toxin were mainly detected in the fresh-cut grass material, while fumonisin and aflatoxin contents were below the detection limits. July and October were the most risky periods for mycotoxins to occur. During the cold temperatures in November and December, the occurrence of mycotoxins in fresh-cut material declined. Although June was a period with low incidence of mycotoxins in green silage, contents of deoxynivalenol and zearalenone in silages from the first cutting exceeded by several times those determined in their biomass collected directly from the field. Moreover, we observed that use of preservatives or inoculants did not prevent mycotoxin production.

## 1. Introduction

Clean and healthy phytomass is a prerequisite for producing high-quality forage. Potential plant contaminants include various epiphyte microflora such as undesirable clostridia (*Clostrium* spp.) and fungi (*Fusarium* spp*.*, *Puccinia* spp.) [1,2]. Development of microscopic fungi may lead to the formation of mycotoxins [3], which are secondary metabolites produced especially by the fungi *Aspergillus*, *Penicillium* and *Fusarium* [4]. Mycotoxins are produced due to interactions and reactions of fungi to environmental conditions [5]. While such production is especially associated with stress caused by extreme weather conditions or damage from insects or animals, mycotoxin contamination of silages is nevertheless associated with failure in silage management practices [6].

Mycotoxins can cause serious health problems in the human population. The incidence of liver cancer caused by aflatoxin is believed to be increasing each year, for example, and up to 28.2% of liver cancer cases may be due to aflatoxin [7]. Mycotoxins also naturally have negative impacts upon livestock, causing alterations in hormonal functions, poor feed utilization, lower rates of body weight gain, and possibly death. Moreover, some mycotoxins may pass into milk, which could represent a risk for the food chain [8,9,10,11].

Preventing the occurrence of mycotoxins in forage should begin in the field, and certain guidelines have been suggested and practices recommended to avoid that. These include the use of varieties or hybrids that are well adapted to the given growing area and that are resistant to fungal diseases [12].

Various grasses are used for grazing and producing stored forages, and considerable differences exist between these grass species. Among those species, *Lolium perenne* is particularly susceptible to fungal infestation. By contrast, *Festulolium* ssp. are considered to be resistant [3]. Interspecific hybrids of *Festulolium* ssp. may combine the endurance of the *Festuca* spp. with the high quality of the *Lolium* spp. *Poa pratensis* and *Festuca rubra* are rhizomatic grasses, which are used to thicken the lower floor stand and contribute to the density of the stands [13].

The aim of the present study was to assess mycotoxin contents in feedstuffs under Central European environmental conditions [14,15] and the potential risks to health safety posed by the presence of mycotoxins in fresh-cut selected forage grass species both during and at the end of the growing season. Furthermore, mycotoxin content was assessed in subsequently produced first-cutting silages with respect to the various species used in this study: *Lolium perenne* (cv. Kentaur), *Festulolium pabulare* (cv. Felina), *Festulolium braunii* (cv. Perseus), and mixtures of these species with *Festuca rubra* (cv. Gondolin) or *Poa pratensis* (Slezanka). The choice of species considered the facts described above, and the various species were either potentially susceptible to diseases or potentially more resistant to disease. When producing silage, a chemical preservative or biological inoculant was applied.

## 2. Experimental Section

### 2.1. Plant Material and Cultivation

**Figure 1:**
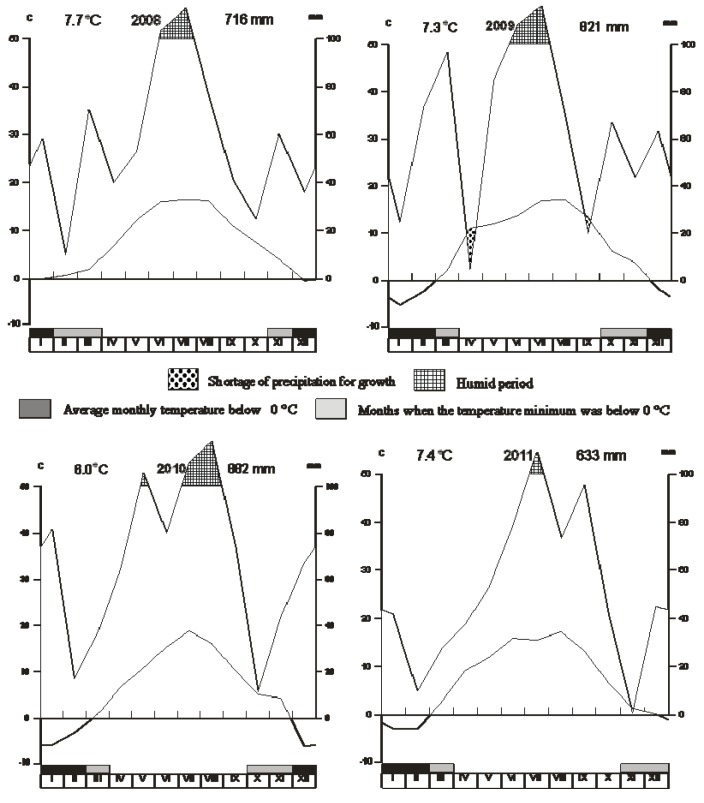
Precipitation and mean temperatures in years 2008–2011 at the Research Station of Fodder Crops, Vatín, Czech Republic.

A small-plot experiment was established in 2007 at the Research Station of Fodder Crops in Vatín, Czech Republic (49°31’N, 15°58’E, 560 m a.s.l.). The climate at the station can be characterized by the 1970–2000 mean annual precipitation of 617 mm and mean annual temperature of 6.9 °C. Figure 1 reports precipitation and mean temperature during the observation years (2008–2011). These data were obtained from a meteorological station situated at the experimental location. The soil type used in our experiments was Cambisol as a sandy-loam on a diluvium of biotic orthogneiss. During the years of observation, the contents of soil nutrients were 89.1 mg·kg^−1^·P, 231.6 mg·kg^−1^·K, and 855 mg·kg^−1^·Ca; pH was 4.76. The experimental plots were fertilized with 50 kg·ha^−1^·N in spring (March). Times of cutting were the beginning of June, end of July, beginning of October, beginning of November and beginning of December. Biomass from the first cutting was ensiled. The experiment was carried out in triplicate. A split-plot design was used with plots of 1.5 × 10 m. The plots were harvested using a self-propelled mowing machine with an engagement width of 1.25 m. Harvested area was 12.5 m^2^. Stubble height was 0.07 m. The grasses were harvested at the earing stage.

### 2.2. Fresh-Cut Material and Silages Preparation

Mycotoxin contents of fresh-cut material and in silages were evaluated. In evaluating fresh-cut material, species was the first factor examined (Table 1). Season was the second factor examined, and it was defined by time of cutting, as follows: beginning of June, end of July, beginning of October, beginning of November and beginning of December. The combined effects of the two factors were also observed. In evaluating silages, grass species was the first factor examined. The second factor was use of inoculant, the groups being untreated, treated with chemical ingredient (formic acid (43% *w*/*w*), propionic acid (10% *w*/*w*), ammonium formate (30% *w*/*w*), benzoic acid (2% *w*/*w*)), and treated with biological–enzymatic inoculant (containing *Enterococcus faecium*, *Lactobacillus plantarum*, *Pediococcus acidilactici*, *Lactobacillus salivarius*, cellulase, hemicellulase, and amylase, with 1 × 10^11^ CFU.g^-1^). The amount of chemical ingredient added was 4 L·t^−1^ of ensiled material and that of biological additive was 10 g·t^−1^. Biological additive was diluted in water at the rate of 2 L·t^−1^. Chemical and biological additives were applied by spraying onto fresh-cut material. During the application, the material was mixed in order to spread the additives evenly. Material for ensiling was harvested from the first cutting in the first week of June. Grasses were allowed to wilt and dry for 20 to 30 h after mowing. The wilted biomass was ensiled in containers with diameter and height 0.15 m and 0.64 m, respectively. Silages were sampled 90 days after closing the containers. Silages were observed in the three years 2008 (1st harvest year), 2009 (2nd harvest year) and 2010 (3rd harvest year). In the fourth harvest year, silages were not produced due to low grass yields.

Silages sampled 90 d after ensiling were assessed for pH, acidity of water extract (AWE), as well as contents of lactic acid (LA), acetic acid (AA), butyric acid (BA) and NH_3_. Values of pH were from 4.05 to 4.26. Content of lactic acid (LA) was from 10.39% to 16.63%, content of acetic acid (AA) was from 1.23% to 3.25%, content of NH_3_ was from 0.1541% to 0.1752% and content of ethanol was from 1.88% to 4.28%.

### 2.3. Mycotoxin Determination

Green forage samples and silages were dried at 60 °C, ground to a particle size of <1 mm, then analyzed for content of the mycotoxins deoxynivalenol (DON), zearalenone (ZEA), fumonisin (FUM), aflatoxin (AFL) and T-2 toxin (T-2) using enzyme-linked immunosorbent assay (ELISA) according to Skladanka *et al*. [16]. ELISA is a competitive, direct enzyme-linked assay for quantitative analysis. The toxin concentration is expressed in parts per billion (ppb). The data were processed statistically using STATISTICA.CZ Version 8.0 (Prague, Czech Republic). The results are expressed as means (×). The results obtained were then further analyzed using ANOVA and Scheffé’s method. Cluster analysis was performed to create graphical representations.

## 3. Results and Discussion

### 3.1. Fresh-Cut Material

**Figure 2:**
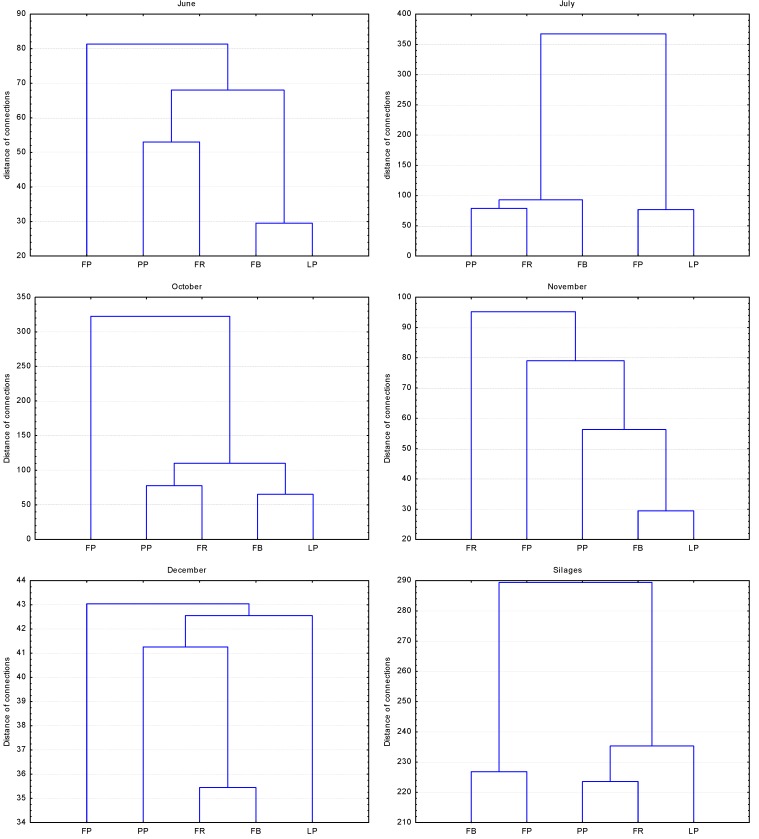
Cluster analysis of evaluated species (Euclidean distance), 2008–2011.

In our study, the mycotoxins DON, ZEA and T-2 were mainly detected. The contents of FUM and AFL were below the limits of detection in the majority of samples. The lowest DON content in fresh-cut material was found in *F. pabulare*, at 31.02 ppb (Table 1). The highest DON content in the fresh-cut material was determined for the mixture with *F. rubra*, at 42.15 ppb. Similarly, the lowest levels of ZEA were determined in the fresh-cut material of *F. pabulare*. Due to high variability among samples, no statistically significant influence of grass species on mycotoxin content was confirmed. There nevertheless was a clear lower tendency for mycotoxins to occur in *F. pabulare*. This is evidenced by the results of the cluster analysis (Figure 2), where *F. pabulare* stands outside a cluster of the other species for June, October and December.

**Table 1:** Influence of species, season (time of cutting) and year on the content (ppb) of deoxynivalenol (DON), fumonisin (FUM), aflatoxin (AFL), zearalenone (ZEA) and T-2 toxin (T-2) in fresh-cut material of grasses.

Factor	DON		FUM		AFL		ZEA		T-2	
	x	S.D.	x	S.D.	x	S.D.	x	S.D.	x	S.D.
**Species**										
*Lolium perenne*	41.03	6.12	<LOQ	-	<LOQ	0,01	17.06	15,52	24.80	5,76
*Festulolium pabulare*	31.02	6.27	<LOQ	-	0.07	0,05	4.95	2,70	24.19	6,27
*Festulolium braunii*	36.98	5.49	<LOQ	-	<LOQ	0,01	36.45	25,05	24.94	5,33
Mixture with *Festuca rubra*	42.15	6.72	<LOQ	-	<LOQ	0,01	47.37	31,50	30.40	6,45
Mixture with *Poa pratensis*	40.19	7.56	<LOQ	-	<LOQ	0,01	48.15	30,99	29.98	6,34
*p*	0.6347		-		0.5288		0.4581		0.7976	
**Season (time of cutting)**										
Beginning June	16.09 ^a^	3,76	<LOQ	-	<LOQ	0,01	1.46	1,43	24.70	6,35
End July	51.90 ^b^	6,55	<LOQ	-	0.09	0,05	61.18 ^a^	33,51	28.49	6,70
Beginning October	41.94 ^b^	5,90	<LOQ	-	<LOQ	0,01	86.55 ^a^	37,83	36.49	5,80
Beginning November	41.58 ^b^	6,99	<LOQ	-	<LOQ	0,01	1.88	1,80	18.25	5,72
Beginning December	39.86 ^a^^,b^	5,97	<LOQ	-	<LOQ	0,01	2.91	1,97	26.39	4,90
*p*	0.0004		-		0.0176		0.0045		0.1112	
**Year**										
2008	37.63 ^a^^,b^	7,65	<LOQ	-	<LOQ	0.01	115.76 ^a^	37,82	34.89 ^a^^,b^	5,26
2009	46.28 ^a^	5,67	<LOQ	-	0.08	0.04	6.15 ^b^	2,50	48.37 ^b^	3,99
2010	47.13 ^a^	3,64	<LOQ	-	<LOQ	0.01	<LOQ ^b^	0,01	5.34 ^c^	3,03
2011	22.06 ^b^	3,78	<LOQ	-	<LOQ	0.01	1.23 ^b^	1,14	18.87 ^a^^,c^	4,51
*p*	0.0019		-		0.0138		0.0000		0.0000	
Species × Season	0.7797		-		0.6986		0.9950		0.9766	
Species × Year	0.9552		-		0.3676		0.6122		0.9906	
Season × Year	0.0000		-		0.0518		0.0000		0.0001	

Mean values in the same column with different superscripts (^a,b,c^) are significant at the *p* < 0.05 level after Scheffé’s method analysis. LOQ = limit of quantification; × = mean; S.D. = standard deviation.

LP = *Lolium perenne*; FP = *Festulolium pabulare*; FB = *Festulolium braunii*; FR = mixture with *Festuca rubra*; PP = mixture with *Poa pratensis*.

Time of cutting especially influenced (*p* < 0.01) the contents of DON and ZEA. Deoxynivalenol content was highest (*p* < 0.05) at the end of July (51.90 ppb). High DON content also remained in October (41.94 ppb) and November (41.58 ppb). In December, DON decreased to 39.86 ppb. Similarly, high ZEA content was found in late July (61.18 ppb), and this culminated in October at 86.55 ppb. The population density of filamentous fungi is known to be positively associated with the senescence process of plants [17], and yet forage from November and December had low levels of ZEA (1.88 and 2.91 ppb, respectively). Reduction of mycotoxins in forage during late autumn and early winter is also evidenced by the analysis for T-2. Inasmuch as the onset of winter could be associated with the death of biomass and the senescent processes would themselves be associated with microscopic fungi capable of producing mycotoxins, one would intuitively expect a rather greater increase of mycotoxins as autumn and winter drew nearer and nearer. In fact, however, the opposite was true. Low temperatures reduce the risk from mycotoxins. It is obvious that the higher humidity of the growing season contributes to the development of mold, but low temperatures inhibit formation of mycotoxins. Fall of the temperature below 5 °C in November and December can lead to a reduction of enzymatic activity of molds and lower production of mycotoxins, which undergo a stress reaction on the lower temperature [18,19,20]. Denijs *et al*., Engels and Krämer, and Behrendt *et al*. had also observed the influence of not only biotic, but also abiotic factors on the production of mycotoxins [17,21,22]. Moreover, higher levels of mycotoxins occurring during winter months were reported by Golinski *et al*. [23]. Forage from the beginning of June is generally characterized by low levels of mycotoxins, and this is especially evident (*p* < 0.01) for DON and ZEA.

The interannual variability of the average DON, ZEA and T-2 contents was significant (*p* < 0.01). In the case of DON, there was an obvious difference (*p* < 0.05) especially between 2010 and 2011. Even more evident differences occurred in ZEA content. While in 2008 ZEA content was 115.76 ppb, it was only 6.15 ppb in 2009 and just 1.23 ppb in 2011. In 2010, ZEA content was even below the limit of detection. Meanwhile, 2010 was characterized by very low T-2 content (*p* < 0.05). There were differences among the evaluated years in terms of total rainfall and its distribution as well as in average annual temperatures and temperature changes.

Moisture, temperature and availability of nutrients and oxygen are among the important factors influencing mold growth [24]. The combination of these factors can have a significant proportionate influence on annual fluctuation in mycotoxin concentrations. In 2008, when the greatest occurrence of mycotoxins in green forage was determined, the highest average annual temperature was measured and precipitation was well distributed within and between months. There was sufficient precipitation for plant growth throughout the year. By contrast, the following years had lower mean annual temperatures and especially the autumn months were characterized by a lack of precipitation. Sometimes, the precipitation curve falls below the curve of temperatures, which indicates lack of moisture for plant growth. This may be reflected also in the growth of mold and subsequent mycotoxin production. Temperature may affect the utilization of certain nutrients in the soil, and in particular phosphorus [25,26]. Reduced nutrients availability can cause plants to have lower resistance to disease and subsequently to be subject to mold development. The year 2008 was among the warmest, and there was a higher incidence of ZEA in the green plant material. In 2009, when there was an obvious drought and rainfall was insufficient for plant growth, higher levels of T-2 were found.

### 3.2. Silages

Grass species had no influence on the content of mycotoxins in silages from the first cutting (Table 2). Differences between species were minimal in the silages produced. There were, however, interesting differences in the contents of mycotoxins between fresh-cut material and silages. The increase in the contents of DON, ZEA and T-2 in silages compared with fresh-cut material is shown in Table 3. DON content in silages increased by as much as 400%. This rise could be due to a higher temperature after closing of the silo containers. Higher temperatures constitute a stress factor that can trigger the production of mycotoxins. After closing the silo containers, the aerobic phase, during which aerobic microorganisms consume the remaining oxygen, produces heat. Mold growth then diminishes during the following anaerobic phase, but the already produced mycotoxins are nevertheless preserved in the silages.

**Table 2:** Influence of species, preservative or inoculant, and year on the content (ppb) of deoxynivalenol (DON), fumonisin (FUM), aflatoxin (AFL), zearalenone (ZEA) and T-2 toxin (T-2) in silages from the first cutting of grasses.

Factor	DON		FUM		AFL		ZEA		T-2	
	x	S.D.	x	S.D.	x	S.D.	x	S.D.	x	S.D.
**Species**										
*Lolium perenne*	141.39	6,29	<LOQ	0,02	<LOQ	0,02	66.07	10,80	20.37	11,29
*Festulolium pabulare*	156.73	19,04	<LOQ	0,02	<LOQ	0,02	47.92	7,99	45.19	25,39
*Festulolium braunii*	143.60	13,24	6.07	6,04	<LOQ	0,01	46.34	8,96	43.04	26,10
Mixture with *Festuca rubra*	161.97	13,86	<LOQ	0,02	<LOQ	0.01	66.89	6,89	38.58	23,88
Mixture with *Poa pratensis*	167.70	15,82	<LOQ	0,02	0.21	0,12	54.46	6,64	19.96	12,1
*p*	0.5142		0.4207		0.2551		0.1577		0.8363	
**Preservative or inoculant**										
Untreated	139.19 ^a^	8,91	<LOQ	0,01	<LOQ	0,01	60.28	6,35	21.81	13,64
Chemical	182.71 ^b^	12,41	<LOQ	3,62	<LOQ	0,01	53.40	8,23	21.64	11,67
Biological-enzymatic	140.93 ^a^	7,22	3.66	0,01	0.14	0,08	55.33	5,23	56.83	20,14
*p*	0.0042		0.3765		0.4899		0.6803		0.2137	
**Year**										
2008	164.61	8,70	<LOQ	0.01	<LOQ	0,01	53.95 ^a^^,b^	5,59	12.67	5,22
2009	156.49	15,19	<LOQ	0.01	<LOQ	0,08	73.24 ^a^	6,94	42.97	11,82
2010	141.73	6,81	3.72	3.62	0.15	0,01	41.81 ^b^	4,68	44.65	23,94
*p*	0.2553		0.3590		0.4037		0.0016		0.2929	
Species × Preservative	0.9502		0.4596		0.3666		0.5255		0.3641	
Species × Year	0.4784		0.4560		0.3753		0.9177		0.8801	
Preservative × Year	0.0004		0.4212		0.3174		0.0362		0.2586	

Mean values in the same column with different superscripts (^a,b,c^) are significant at the *p* < 0.05 level after Scheffé’s method analysis. × = mean; S.D. = standard deviation.

The highest content of mycotoxin generally, and of DON in particular (167 ppb), was found in the mixture with *P. pratensis*. Charmley *et al*. have reported that DON may be passed to milk when its content in feedstuffs reaches the level of 6 mg·kg^−1^ [8]. The European Commission advisory guideline for DON is 5 mg·kg^−1^ of dry matter (Commission Recommendation of 17 August 2006 on the presence of DON, ZEA, ochratoxin A, T-2 and HT-2 toxins, and fumonisins in products intended for animal feeding (2006/576/EC)). Zearalenone content increased by as much as 868% in silage from *F. pabulare*. The highest ZEA content was determined in the silage mixture with *F. rubra* (66.89 ppb). The guidance value for ZEA in Europe is 500 µg·kg^−1^ of dry matter. According to D’Mello, ZEA in concentrations ranging from 0.2 to 1.0 mg·kg^−1^ is even toxic to rodents [27]. It is advised not to use for feeding purposes forage with ZEA content higher than 0.5 mg·kg^−1^ [28]. Aside from FUM and AFL, for which no differences between the fresh-cut material and silages were found, the smallest changes after ensiling were recorded for T-2. T-2 content in silages increased by a maximum of 86.8% in the case of *F. pabulare*.

**Table 3:** Differences (%) in content (ppb) of deoxynivalenol (DON), zearalenone (ZEA) and T-2 toxin (T-2) between fresh-cut material and grass silages. FCM = fresh-cut material, S = silages.

Factor	DON			ZEA			T-2		
	FCM	S	*Rel.%*	FCM	S	*Rel.%*	FCM	S	*Rel.%*
*Lolium perenne*	41.03	141.39	*344.6*	17.06	66.07	*387.3*	24.80	20.37	*82.1*
*Festulolium pabulare*	31.02	156.73	*505.2*	4.95	47.92	*968.0*	24.19	45.19	*186.8*
*Festulolium braunii*	36.98	143.60	*388.3*	36.45	46.34	*127.1*	24.94	43.04	*172.6*
Mixture with *Festuca rubra*	42.15	161.97	*384.3*	47.37	66.89	*141.2*	30.40	38.58	*126.9*
Mixture with *Poa pratensis*	40.19	167.70	*417.3*	48.15	54.46	*113.1*	29.98	19.96	*66.6*

The increase of mycotoxins in silages was in some cases very significant. Ensiling is a process whereby lactic acid bacteria ferment simple sugars and produce acids. This reduces the pH and consequently there is diminished growth of undesirable microorganisms. The increase of mycotoxins within the silages was probably caused by the production of mycotoxins during wilting of the cut grass and the first phase of aerobic fermentation. Because an anaerobic environment reduces the growth of fungi, ensiling is from this perspective is an effective strategy to prevent the production of mycotoxins [6]. Material for producing silage is contaminated with mycotoxin-producing fungi already in the field, and consequently the feeding safety continues to diminish at least through the first several hours after the start of ensiling. Our findings support earlier observations that DON, ZEA and other *Fusarium* mycotoxins are produced in silages [24]. In any case, our results indicate that mycotoxins were generally not degraded by the ensiling process. Nevertheless, there are other studies demonstrating potential for strongly reducing mycotoxins production during the ensiling process by using, for example, inoculants [29,30].

Cluster analysis (Figure 2) in relation to the silages shows, on the one hand, a similarity between the intergeneric hybrids (*F. pabulare* and *F. braunii*) and, on the other hand, a cluster of *L. perenne* and both mixtures including *F. rubra* or *P. pratensis*.

The preservatives used in our study did not prevent mycotoxin production although these materials are commonly used by farmers with the aim of improving the ensiling process. In the case of DON, the addition of organic acids even led to an increase (*p* < 0.05) in the content. It is precisely the addition of organic acids, and in particular propionic acid, which has antifungal effects [31]. Nevertheless, acids and inoculants have no effect on mycotoxins that already have been synthesized.

We observed an effect of year on ZEA content in silages (*p* <0.01). The lowest ZEA content (*p* < 0.05) was found in silages during 2010, in which year ZEA concentrations were similar to those in fresh-cut material.

## 4. Conclusions

Mycotoxins are secondary metabolites with harmful effects on mammals. Their concentrations are therefore monitored and their effects intensely studied in fresh material. In this study, we investigated several factors influencing the content of these secondary metabolites in fresh-cut material and silages prepared from various grass species. It can be concluded that low temperatures can be beneficial for inhibiting the production of mycotoxins. This is well documented by the above mentioned results, however, these conditions can only be taken into account in some part of Europe, mainly in the middle and northern regions. On the other hand, these places are beneficial for the growing of the mentioned species, because they are also resistant to that environment together with the lower content of mycotoxins. It should also be taken into account that the processing of green material for silage can itself contribute to increasing mycotoxin concentrations.
